# The Effectiveness of Fork-Mashable Dishes in Managing Older Patients with Mastication and Swallowing Impairments

**DOI:** 10.3390/foods14101723

**Published:** 2025-05-13

**Authors:** Kovan Ismael-Mohammed, Mireia Bolivar-Prados, Laura Laguna, Adrian Nuñez Lara, Marta Cera, Paula Viñas, Pere Clavé

**Affiliations:** 1 Gastrointestinal Physiology Laboratory, Hospital de Mataró, Universitat Autònoma de Barcelona, 08304 Barcelona, Spain; 2 Institute of Agrochemistry and Food Technology (IATA, CSIC), 46980 Valencia, Spain; 3 Centro de Investigación Biomédica en Red de Enfermedades Hepáticas y Digestivas (Ciberehd), 08304 Barcelona, Spain

**Keywords:** fork-mashable dishes, texture E, textural properties, swallowing safety, oral residues, pharyngeal residues

## Abstract

Background: The optimal textural properties and therapeutic effects of fork-Mashable dishes for hospitalized older patients with oropharyngeal dysphagia (OD) have not been adequately defined. Objectives: This study aimed to (a) quantify the textural properties of six fork-Mashable dishes (British Dietetic Association (BDA) texture E; International Dysphagia Diet Standardization Initiative (IDDSI) level 6), (b) assess the impact of oral processing on texture, and (c) evaluate their safety and efficacy in older patients with OD. Materials and Methods: Twenty patients (85 ± 4.51 years) consumed six 30 g dishes. Oral processing was analyzed using surface electromyography (EMG), texture was measured pre- and post-oral processing, and swallowing safety was assessed using the volume–viscosity swallowing test (V-VST). Results: Although all the dishes met the BDA E/IDDSI 6 descriptors, significant differences were found in both safety (ranging from 50–100%, *p* < 0.05, for four dishes vs. thin liquids) and efficacy outcomes (oral residues 60–100%; pharyngeal residues 20–70%; *p* < 0.05 for all dishes vs. liquids and French and zucchini omelets vs. 250 mPa·s). Textural characteristics showed wide variability. Oral processing reduced MF but increased adhesiveness, except for in French omelet and pollock fish. The patients required 29–31 mastication cycles over 21–28 s. The post-oral texture also varied significantly across dishes. Conclusions: The therapeutic effect of our diets was independent of the BDA or IDDSI levels, with great variations in safety and swallowing efficacy. Textural properties, oral processing behavior, and individual patient responses played decisive roles. Variations in maximum force and adhesiveness during oral processing were crucial for the therapeutic effect, as indicated by the principal component analysis (PCA) correlation.

## 1. Introduction

One of the side effects of aging is a reduction in mastication and swallowing abilities [1,2], which can cause both mastication impairments and oropharyngeal dysphagia (OD). Both of these conditions can reduce the quality of life, nutrition status, and dehydration status and cause aspiration pneumonia [3,4].

Several countries have reported large numbers of aging people with mastication impairments. A study carried out by Dias da Costa et al. in 2010 showed that the percentage of older people with poor mastication abilities was 49.7% [5]. Another study carried out in Mexico showed that mastication difficulties (temporomandibular joint disorder) among people aged between 60 and 69 years were 33.1% [6]. Dibello et al. (2021) said that the reduction in the function of oral motor skills is a factor that contributes to frailty in older people [7]. Regarding swallowing impairments, the prevalence ranged from 10% to 33% [8], and 16.6% of those affected were living independently and were between 70 and 79 years old. This percentage rises to 33% as age increases. This percentage of aged people who suffer swallowing impairment could reach 47% when considering people who are hospitalized and or admitted to the hospital due to various reasons [9].

To help people with mastication impairments and OD who cannot consume regular food items, many health centers provide texture-modified diets. One of these health centers is Hospital de Mataro, which adopted the Triple Adaptation [10] and prepared dozens of recipes in the hospital kitchen according to a qualitative manual provided for people with swallowing and mastication disorders, adapted from the British Dietetic Association (BDA) manual [11]. There are several other qualitative classification system manuals available for texture-modified diets (TMDs), such as the International Dysphagia Diet Standardization (IDDSI) [12], the National Dysphagia Diet (NDD) [13], the Japanese Dysphagia Diet (JDD), and Smile Care [14,15]. As all the classifications depend on the qualitative measurements for preparation, they have been proved not to be precise when the measurement is carried out using SI units [16,17]. The IDDSI scale has eight levels of classifications, starting with 0, which corresponds to liquid, up to level 7, which is normal food. The BDA designed a scale of four levels, B, C, D, and E, with B being thin puree; C, thick puree; D, pre-mashed, and E, fork-Mashable diets. The NDD also had four groups for solid food, starting from level 1 (dysphagia pureed), level 2 (dysphagia mechanically altered), level 3 (dysphagia advanced), and level 4 (regular food). Both the BDA and the NDD have since adopted the IDDSI scale. Japan has two systems of TMD classification, the Japanese Dysphagia Diet (JDD) 2021 and the Smile Care system: The JDDS also has seven types of TMDs, starting from Code 0t, representing a thickened liquid; Code 0j, which is jelly-like; Code 1j, 2-1, and 2-2, are puree-like; Code 3 is a solid that can be crushed without using the teeth; and finally, Code 4, which requires some mastication and cannot be crushed between the tongue and palate [14]. The Smile Care system classifies the food based on people’s mastication abilities and gives them a color code: blue for healthy people, yellow for people with mastication problems, and red for people with swallowing problems [15].

TMDs are thus being used for both mastication and swallowing impairments; however, the question still arises: what are the optimal levels of the textural properties of those foods for safe and efficient swallowing? One widely used technique for food evaluation is the texture profile analysis (TPA), which was first described in 1963 and has been used as a standard method in the food industry. However, while the TPA can provide important information on textural properties, a standardized protocol is needed to evaluate food in the context of dysphagia and mastication impairment management. To achieve this goal, we examined biomechanical mastication parameters, swallowing safety, and efficacy, using tests of both mastication and swallowing solids (adapted from TOMASS) to evaluate mastication and the volume–viscosity swallowing test (V-VST) for impaired swallowing evaluation. The TOMASS test was developed using a cracker to complete the test [18]. Following this method, the volunteer eats the cracker as fast as they can, and then the solid oral preparation is evaluated. However, in this study, we adapted the original TOMASS and used our fork-Mashable dishes to evaluate the parameters. In addition, electromyography was used for muscle activity, including mastication cycles, frequency, and time. The V-VST is a clinical tool to assess dysphagia with high accuracy for both safety and efficacy evaluation during swallowing [19,20], demonstrating a sensitivity to OD of 93.17%, a reliability of 81.39%, and a Kappa = 0.77.

Despite fork-Mashable dishes being used widely in different health centers to manage OD, optimal textural properties, characteristics after oral processing, the safety and efficacy of swallowing, palatability, and whether they meet the preferences and needs of individuals with dysphagia have yet to be studied. The evaluation of their textural properties, oral processing dynamics, and the biomechanical aspects of mastication and the safety and efficacy of the swallowing process of fork-Mashable dishes is necessary to build a comprehensive method to assess their impact on patients with OD and mastication disorder. This study aims to fill this research gap and assess whether these dishes can be swallowed safely and efficiently and whether they meet the needs of older individuals.

The main objectives of this study were as follows: (a) to quantify the basal textural properties of six fork-Mashable foods (BDA texture E, IDDSI level 6) using the International System of Units (SI); (b) to determine the biomechanics of oral processing and the modifications it produces on texture, by a modified TOMASS using EMG; and (c) to evaluate the safety and efficacy of swallowing these fork-Mashable dishes in older patients with swallowing and mastication disorders using the V-VST.

## 2. Materials and Methods

### 2.1. Material

#### 2.1.1. Texture-Modified Deits

The caterers, Serhs Food SA (Mataro, Spain), provided six dishes that were cooked in the hospital kitchen, as explained in a previously published paper [16] and were examples of the dishes prepared for patients with dysphagia. These dishes were French omelet, zucchini omelet, stewed turkey, red lentils, potato, and pollock fish. The dishes were all classified as texture E based on the qualitative BDA classification (level 6 according to the IDDSI). (See Supplementary Figure S2).

#### 2.1.2. Fork-Mashable Dishes

Characteristics and texture checks for the fork-Mashable qualitative classification were the same as in our previous study [16].

#### 2.1.3. Composition of Fork-Mashable Dishes

Table 1 below lists the dishes’ ingredients, weight per recipe, proteins, and kcals.

#### 2.1.4. Participants

We recruited 20 participants from the database of the hospital, 50% males, with an average age of 85 ± 4.51 years. The patients had already been diagnosed with clinical complaints of mild swallowing disorders, using the V-VST test, and with complaints of mastication impairments, identified by adapting the self-reported test developed by Ludis so et al. (2007) [21]. In this test, the participants were asked two questions: 1. Do you experience difficulties chewing? 2. Did you change your alimentary habits because of such a difficulty? They were also asked a question about their dental status: Do you have any of these? Bridges, Crowns, Dentures, Toothlessness? All the questions were asked during a phone call and were re-asked during the visit.

#### 2.1.5. Oral Moisture Levels and Hand Strength

On the day of the session, oral moisture and hand strength were measured using the oral-moisture-checking device (Mucus©, approval number: 22200BZX00640000, Life Co., Ltd., Saitama, Japan), with the measurement being taken 10 mm from the tongue tip. The hand dynamometer Grip-A Takei was used to measure the handgrip force (Takei Scientific Instruments Co., Ltd., Niigata, Japan).

### 2.2. Study Design

The texture properties of the pre-oral and the post-oral processing (bolus ready to swallow (BRS) phase, along with the oral processing effect on the 6 dishes, were performed using a similar method as to that published in our previous publication [16].

**Figure 1:**
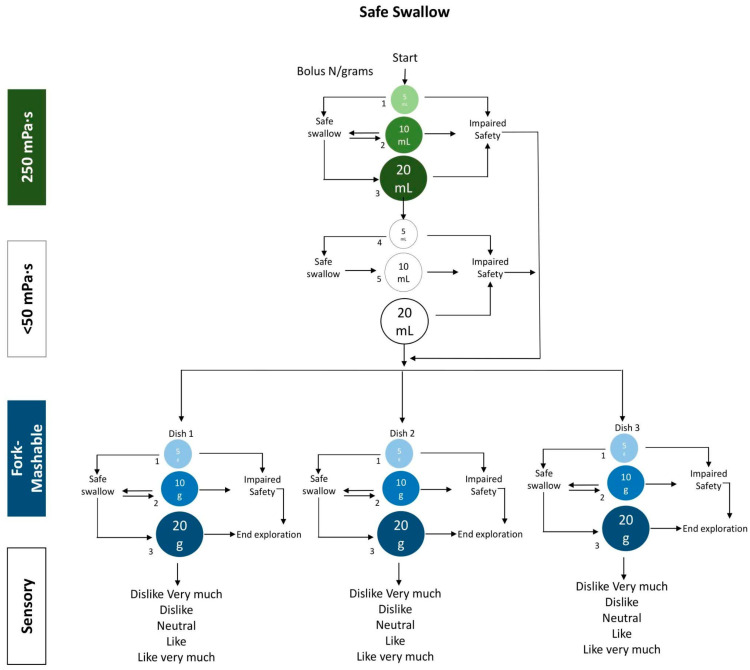
The V-VST-adapted algorithm used to evaluate the safety, efficacy, and palatability of the fork-Mashable dishes.

- The TOMASS test was adapted by us and was used to count the number of masticatory cycles, time, and frequency for each dish until the bolus was ready to swallow, according to our previous studies [16].
- The standard volume–viscosity swallowing test (V-VST) confirmed swallowing difficulties; both processes took place during the session.
- To verify the dishes’ safety and efficacy when swallowed, an adapted V-VST was used to compare both the liquid and the 250 mPa·s viscosities. Three boluses of 5 g, 10 g, and 20 g per fork-Mashable dish were tested (instead of the pudding series). Each participant tried three dishes, so in total, each participant carried out nine replicates (See Figure 1).
- Furthermore, oral moisture levels were measured in 15 participants using the Mucus© device [22] in specific oral regions, including approximately 10 mm from the tip of the tongue. Additionally, handgrip strength was assessed in these participants following the methodology described by Laguna et al., 2016 [23]. This study protocol received approval from the Ethics Committee of the Consorci Sanitari del Maresme, under code 63/22.

### 2.3. Sample Size

The sample size was determined based on our previous study, and the sample size was the minimum needed to determine significant differences. There was an alpha risk of 0.05 and a power of 0.80 and a reduction of the proportions from 0.99 to 0.25 with a 20% dropout rate expected.

### 2.4. Methodology

#### 2.4.1. Textural Properties

For this study, a TPA using a texture analyzer TA.XTplus for both pre-oral processing and the boluses produced after oral processing (Stable Micro Systems, Godalming, UK) was applied to characterize the dishes of the fork-Mashable texture based on the protocol developed in a previous study [16]. The software version of the exponent was 7.0.6.0, and stable Microsystems were used for the data collection. The weight loaded on the cell of the texture analyzer was 5 kg, and 0.049 N of trigger force was used. The recipient used acrylic with a diameter of 49.5 mm and a height of 72 mm, combined with an aluminum probe, which was cylinder-shaped (36 mm diameter). A 30 g sample of each dish was added to the recipient at a temperature of 40–50 °C at the pre-oral stage only, without standardizing the sample height or geometry due to the varying consistencies and compositions of the hospital-prepared dishes. We did not standardize initial sample height or geometry, so the compression strain was approximately 75% based on the initial sample height (about 15–20 mm). At the post-oral stage, after the bolus was collected, it was also introduced to the same recipient with no alteration to its temperature. The test speed used was 1 mm/s. The compression stopped at 5 mm from the bottom, and the probe was immediately moved backwards. The maximum force (hardness, the peak force of first compression), cohesiveness (the ratio of the 2nd positive area/1st positive area), and adhesiveness (first negative area) were the parameters that were obtained from the curve of this test. The results were presented in SI units.

#### 2.4.2. Biomechanical Assessment of Oral Processing

Biomechanical assessments of oral processing were performed using a modified TOMASS method [18]. The participants were seated in a comfortable position while electrodes were attached to the masseter and submental muscles, along with a placement on the bone structure on either the right or left side. An accelerometer was positioned under the Adam’s apple to capture the relevant motion data. The sessions were recorded with a Logitech C920 PRO HD Webcam (Logitech C920 PRO HD Webcam (Logitech Inc., Newark, CA, USA) to facilitate a detailed post-analysis. Using a normal spoon, the participants were provided with food and instructed to process it in their mouth until the bolus was ready to swallow (BRS). The participants were asked to spit out the bolus as outlined in Section 2.2. The Electromyography data and video footage were analyzed Biopac Systems MP150 (Biopac Systems, Inc., Goleta, CA, USA) with Biopac software version 5.0. The parameters measured were the mastication cycles, meaning the total number of complete jaw movements, including the opening and closing actions needed to prepare the bolus for swallowing; the mastication time, meaning the total duration required to perform all the mastication cycles and prepare the given food quantity to achieve BRS; and the mastication frequency, meaning the speed at which mastication cycles occurred, expressed in cycles per second.

#### 2.4.3. Volume–Viscosity Swallowing Test (V-VST)

The adapted V-VST was used to assess the safety and efficacy of the fork-Mashable dishes, as stated in Section 2.2 (Study Design) using the following clinical parameters.

Signs of impaired swallowing efficacy.

Swallowing efficacy was assessed by monitoring the following clinical signs:

- Labial seal: check if any portion of the bolus has escaped from the labial seal.
- Oral or pharyngeal residue: checking the mouth for any food residues (oral residues) and asking participants if they felt any food stuck in their throat or any urge to swallow again (pharyngeal residues) after each swallow.
- Piecemeal deglutition: checking for multiple swallows for one bolus.

Signs of impaired swallowing safety.

The safety of swallowing was checked via the following clinical signs.

- Voice changes: voice change was detected by asking participants to say their name before swallowing and then after each swallow.
- Coughing: a sign indicating a risk of aspiration.
- Oxygen saturation: The baseline of the oxygen saturation was measured two minutes before the test, using a finger pulse oximeter placed on the right index finger. Oxygen was recorded after swallowing, and a reduction in oxygen saturation of ≥3% compared to baseline was considered a sign of aspiration.

For this study, an adapted V-VST protocol was implemented, changing the 800 mPa.s for texture E (Figure 1). The participants were provided with three bolus volumes (5 mL and or g, 10 mL and or g, and 20 mL and or g) for each of two viscosities, using grams to measure the fork-Mashable dishes:

1. Liquid (<50 mPa·s).
2. 250 mPa·s thickened liquid. A gum-based thickener was used based on the predetermined weight in g mixed with 100 mL of water. The viscosity used in the V-VST was determined at a shear rate of 50 s^−1^, to approximate the conditions in the oral cavity. All the viscosity measurements were conducted at a controlled temperature of 25 °C.
3. Texture E/IDDSI 6.

Sensory assessment

The participants were asked to rate their overall liking and specific attributes of the dishes, which included the appearance, texture, smell, and taste, by using a 5-point, adapted, hedonic scale with faces:

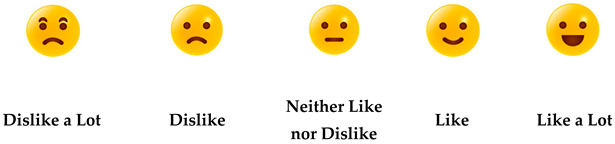



These ratings were collected immediately after the participants swallowed the fork-Mashable samples during the V-VST test to capture their sensory impressions and preferences.

#### 2.4.4. Analysis of Oral Processing

The textural properties percentages were calculated using the following formula:

$$\%\;Maximum\;Force=\frac{\left(Maximum\;force\;at\;pre\;oral)-(Maximum\;force\;at\;post\;oral\right)}{\left(Maximum\;force\;at\;pre\;oral\right)}\times 100$$

$$\%\;Adhesiveness\;or\;coehsivneess=\frac{\left(Adhesiveness\;or\;coehsivneess\;at\;pre\;oral)-(Adhesiveness\;or\;coehsivneess\;at\;post\;oral\right)}{\left(Adhesiveness\;or\;coehsivneess\;at\;pre\;oral\right)}\times 100$$

### 2.5. Data Management and Statistical Analysis

The data of the present study are shown as means ± SD. A total of 120 samples and replicates were made from the six fork-Mashable dishes, including pre- and post-oral processing stages. The comparison was performed among the post-oral processing values produced, using a Kruskal–Wallis test with *p* < 0.05 to determine the differences in terms of textural properties (maximum force, cohesiveness, and adhesiveness). A Z test was used to compare the percentages of the V-VST parameters (both safety and efficacy) to compare against liquid and 250 mPa.s. A principal component analysis (PCA) was used to summarize all the previous parameters and understand the interplay among them. These textural properties were not directly compared statistically across dishes in the pre-oral stage due to the differences in geometry, nor were they compared for the pre-oral vs. post-oral values; the data highlight the variability in texture before oral processing, potentially influencing bolus formation and swallowing safety.

## 3. Results

### 3.1. Moisture and Hand Strength

The results of the mouth moisture levels and hand grip strength are presented in Table 2.

### 3.2. Textural Properties

#### 3.2.1. Pre-Oral Characteristics

The pre-oral processing textural properties for texture-modified diets (Table 3) showed that the maximum force ranged from 1.03 ± 0.48 N for potato to 1.7 ± 0.9 N for zucchini omelet (39.41% of variation). Adhesiveness ranged from 0.02 ± 0.01 for zucchini omelet to 0.9 ± 0.5 for red lentils (97% of variation), while cohesiveness varied between 0.3 ± 0.17 N for potato and 0.8 ± 0.05 N for French omelet (59.30% of variation).

#### 3.2.2. Bolus Ready to Swallow

Post-oral (bolus ready to swallow) characteristics: For the post-oral processing properties (Table 3), the maximum force ranged from 0.54 ± 0.16 N for French omelet to 2.12 ± 0.87 N for stewed turkey (74.52% of variation). Significant differences were observed between French omelet and potato (*p* = 0.024), red lentils (*p* = 0.024), and stewed turkey (*p* = 0.003). Red lentils also differed significantly from stewed turkey (*p* = 0.007) and pollock fish (*p* = 0.002). Adhesiveness values varied from 0.01 ± 0.005 for pollock fish to 1.53 ± 0.45 for potatoes (99.34% of variability). Statistical differences were found between French omelet and potato (*p* = 0.002), pollock fish (*p* = 0.009), red lentils (*p* = 0.002), and stewed turkey (*p* = 0.002). Additional significant comparisons included potato versus pollock fish (*p* = 0.002) and zucchini omelet (*p* = 0.003), as well as pollock fish versus red lentils (*p* = 0.002) and zucchini omelet (*p* = 0.01). Cohesiveness ranged between 0.52 ± 0.11 for zucchini omelet and 0.75 ± 0.04 for red lentils (30.66% variability. Significant differences were observed between French omelet and stewed turkey (*p* = 0.005), pollock fish (*p* = 0.007), red lentils and stewed turkey (*p* = 0.002), and red lentils versus zucchini omelet (*p* = 0.007).

#### 3.2.3. Oral Processing Effect

The oral processing (OP) effects on the TMDs varied across the maximum force, adhesiveness, and cohesiveness.

For the maximum force, the greatest reduction was observed in French omelet (51.75%) and pollock fish (38%). Red lentils and zucchini omelets also showed reductions of 28% and 16.20%, respectively. In contrast, stewed turkey experienced an increase in the maximum force (−51%), indicating greater resistance post-oral processing, while potato showed a minimal reduction (7.6%).

Regarding adhesiveness, the reductions were found in French omelet (88%) and pollock fish (83%), with red lentils showing a smaller reduction (32%). However, zucchini omelet, stewed turkey, and potato showed notable increases in adhesiveness post-oral processing, with values of 92%, 75%, and 86%, respectively.

**Figure 2:**
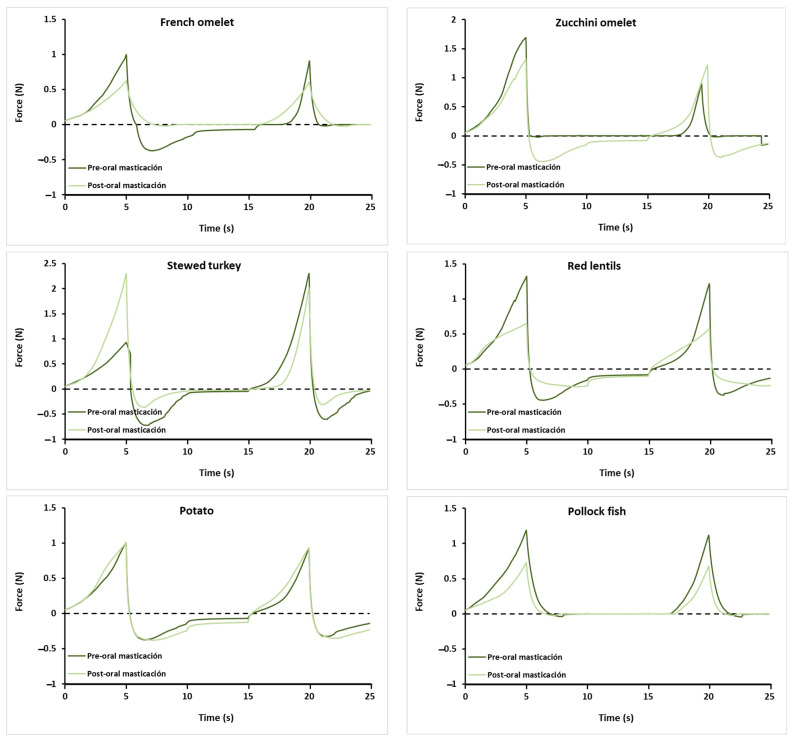
Examples of the curves obtained with the texture profile analysis test of the 6 fork-Mashable samples pre- and post-oral processing.

For cohesiveness, the largest reduction was observed in potatoes (−85.71%), followed by red lentils (−38.89%) and stewed turkey (−20.45%). In contrast, French omelet and zucchini omelet showed minor reductions of −19.77% and −23.53%, respectively, while pollock fish exhibited a slight reduction of −7.58%. (see Table 3, Figure 2).

### 3.3. Biomechanics of Mastication

Table 4 summarizes the mean values of the biomechanical mastication work for various food items. The stewed turkey exhibited the highest mean mastication cycles (MCs) at 33 ± 19.60, accompanied by a time of 24.89 ± 0.32 s and a frequency (Fq) of 1.25 ± 0.31. No significant differences were presented when all the dishes were compared against each other concerning MCs, Time, and Fq.

### 3.4. Safety of Swallowing

**Figure 3:**
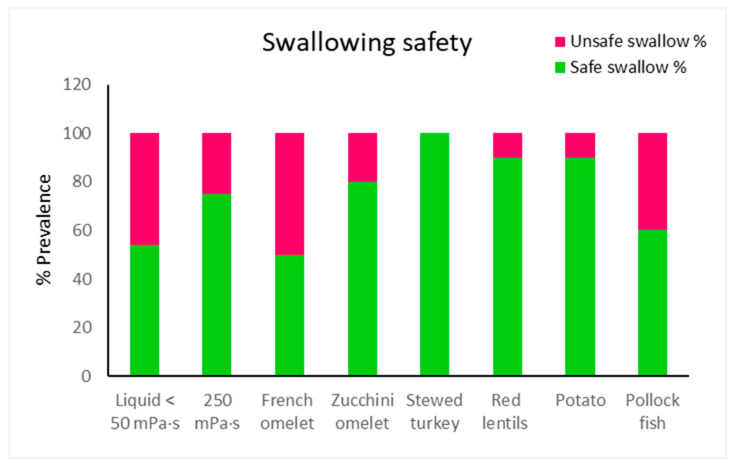
Percentage of patients with safe and unsafe swallows by viscosity and dishes.

Figure 3 presents the mean percentages of safe and unsafe swallows for various TMDs and liquid viscosities. Stewed turkey showed the highest percentage of safe swallowing in 100% of patients, indicating that it is entirely safe for consumption. Conversely, French omelet exhibited the lowest percentage of safe swallowing in only 50% of patients. Statistically significant safety improvements (*p* < 0.05) were observed for zucchini omelet, stewed turkey, red lentils, and potato, compared to liquid, while French omelet and pollock fish showed no significant difference. Against 250 mPa·s, French omelet and pollock fish showed significantly lower safety (*p* < 0.05), whereas stewed turkey, red lentils, and potato had (*p* < 0.05). Zucchini omelet showed no significant difference. (See Supplementary Figure S1).

### 3.5. Efficacy of Swallowing

**Figure 4:**
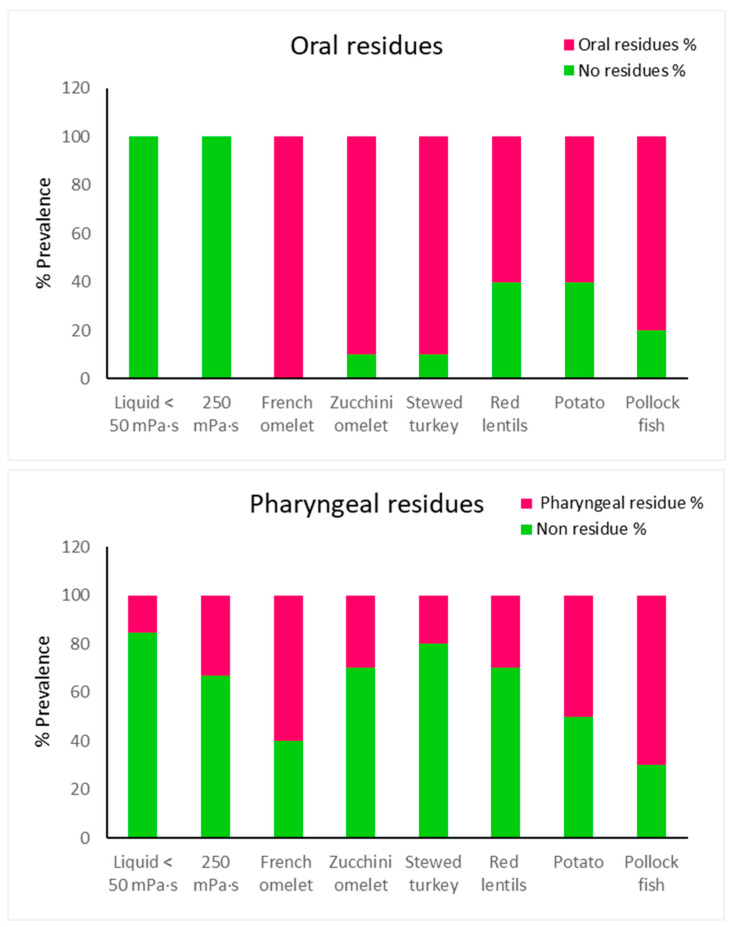
Percentage of oral and pharyngeal residues and non-residues by viscosity and dish.

Figure 4 summarizes the mean values of oral and pharyngeal residues for various texture-modified foods. Statistically significant increases in oral residues (*p* < 0.05) were observed for French omelet, zucchini omelet, stewed turkey, red lentils, potato, and pollock fish compared to liquid and 250 mPa·s.

Regarding pharyngeal residues, French omelet and zucchini omelet (*p* < 0.05) had significantly higher residues compared to liquid, while stewed turkey showed no significant difference. Against 250 mPa·s, French omelet (*p* < 0.05) had significantly higher pharyngeal residues, whereas zucchini omelet showed no significant difference (See Supplementary Figure S1).

### 3.6. Sensory

The sensory evaluation of fork-Mashable dishes revealed varied acceptance levels. Overall, the participants’ liking showed that stewed turkey and red lentils scored highest at 50% Like, while zucchini omelet and potato had 0% for Like a Lot. In terms of appearance, potatoes received the highest rating at 56%, while stewed turkey had a “Dislike” at 33%. For texture, potato excelled with 78% Like, whereas stewed turkey achieved 50% Dislike. Regarding smell, French omelet was favored with 70% Like, while zucchini omelet recorded 22% Dislike. Finally, for the aftertaste, stewed turkey had 50% Like, and red lentils garnered 75% Neither Like nor Dislike. These findings underscore the significance of texture and smell in the acceptance of fork-Mashable dishes (see Table 5).

### 3.7. Principal Component Analysis (PCA)

**Figure 5:**
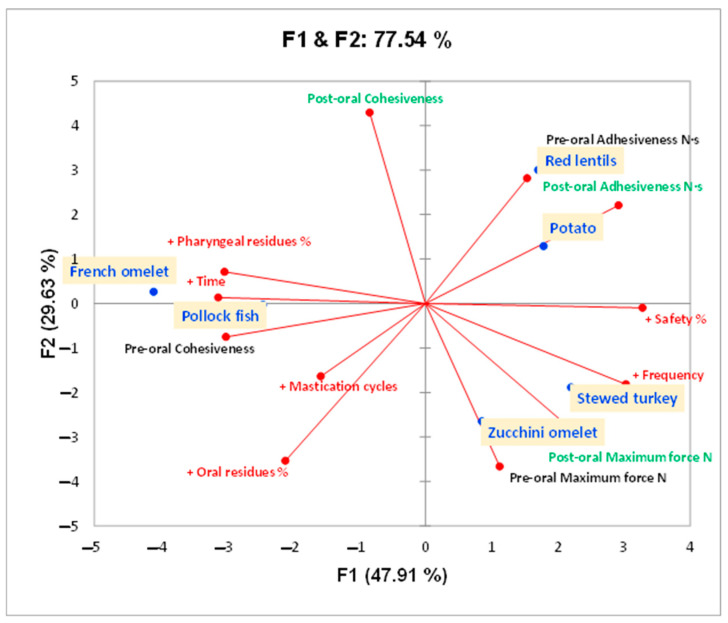
The principal component analysis (PCA) plot illustrates the relationships between different fork-Mashable dishes based on their textural and biomechanical properties, as well as their safety and efficacy percentages, during oral processing and swallowing. The orange squares represent the dishes tested. Variables shown in green indicate measurements taken after oral processing, while those in black represent pre-oral processing variables. Red labels represent additional variables. “+” indicates an increase in values.

A PCA was conducted to summarize all the data obtained. The PCA in Figure 5 showed 77.54% for the total variable variation. The F1 (first component) represents 47.91% of the variability and separates French omelet and pollock fish from the rest of the dishes, due to the high oral and pharyngeal residue, time, and mastication cycles needed for bolus preparation and the high cohesiveness at the pre-oral stage. The F2 (second component) separated both stewed turkey and zucchini omelet from the rest of the dishes due to the high maximum force at both the pre- and post-oral stages, safety, low pharyngeal residue, and frequency of masticatory cycles. Finally, both the maximum force and the adhesiveness of the fork-Mashable diets are the textural properties responsible for the safe swallowing of red lentils, potato, stewed turkey, and zucchini omelet; all of them are projected on the right side of PCA 1 (see the figure below PCA 1, right side).

## 4. Discussion

This study evaluated the textural properties, oral processing behavior, and swallowing safety and efficacy of six fork-Mashable dishes provided to patients with mastication and swallowing difficulties at Mataró Hospital. All the dishes met the BDA texture E and IDDSI level 6 descriptors. Although all the dishes met BDA E/IDDSI 6 descriptors, significant differences were found in both their safety (ranging from 50–100%, *p* < 0.05, for four dishes vs. thin liquids) and efficacy outcomes (oral residues 60–100%, pharyngeal residues 20–70%, *p* < 0.05 for all dishes vs. liquids, as well as for French and zucchini omelets vs. 250 mPa·s). Pre-oral texture measurements showed variability in the maximum force (1.03–1.70 N), cohesiveness (0.35–0.86), and adhesiveness (0.02–0.99 N·s). After oral processing, the maximum force decreased for most of the dishes, while the adhesiveness increased in the potato, zucchini omelet, and stewed turkey. The French omelet and the pollock fish were the only dishes where adhesiveness decreased.

The patients required 29–33 mastication cycles over 21–28 s, with no significant differences across the dishes. The swallowing safety improved significantly (*p* < 0.05) for four dishes (stewed turkey, red lentils, zucchini omelet, and potato), achieving 80–100% safety, compared to 54% for thin liquids and 75% for 250 mPa·s. The oral residue significantly increased for all the dishes versus liquids (*p* < 0.05), ranging from 60% to 100%, while the pharyngeal residue ranged from 20% to 70%, with significant differences observed for the French and the zucchini omelets versus thin liquid (*p* < 0.05) and for the French omelet versus 250 mPa·s (*p* < 0.05).

All the dishes in the study were designed previously by a multidisciplinary team of physicians, dietitians, and nutritionists when the qualitative descriptors were adapted to the hospital kitchen, as described in the study on the Triple Adaptation for the Mediterranean Diet [10]. The focus, at that time, included three main keys to providing safe food to patients: (a) rheological adaptation to optimize texture and consistency for safe mastication and swallowing, (b) nutritional adaptation to meet the water, calorie, and protein needs of patients, and (c) organoleptic adaptation to enhance palatability and patient compliance.

Regarding the effect of oral processing on the textual properties of the fork-Mashable dishes, the impact varied, probably due to the ingredients of the dishes and their preparation [16]. The reductions in the textural properties due to oral processing identified were maximum force 7.6–51%, cohesiveness 7–23%, and adhesiveness 88%, but adhesiveness also increased for several dishes by 32–92%. The reduction in the maximum force during oral processing observed across all the dishes is expected, as mastication naturally breaks down the food into smaller, softer particles to prepare it for swallowing [24]. Larger reductions in dishes like the French omelet and the pollock fish may reflect their softer, less cohesive initial structure, while the minimal reductions in the potato and the zucchini omelet suggest these dishes maintain a firmer structure during mastication, likely due to the presence of starch or fibrous components. While the cohesiveness generally decreased during oral mastication, the extent of its reduction was relatively small compared to the maximum force and adhesiveness observed in dishes like the potato and the zucchini omelet, likely resulting from the gelatinization of starches during mastication, which makes the bolus stickier. This might hinder efficient clearance from the oral cavity, contributing to higher residue levels. Significant reductions in cohesiveness in dishes like pollock fish and the French omelet may be due to their high moisture content, which reduces stickiness as the food breaks down.

An adapted TOMASS was used, following the same protocol used in our earlier study [16,17], to study the oral phase properties of fork-Mashable dishes (BDA texture E). In the earlier study, a variation was noted in parameters MC, time, and FQ by 59%, 56%, and 33%, respectively, ranging between 19 and 38 for MC, 18 and 36 s for time, and 1.1 and 1.68 for fq [16]. In this study, the variation was lower: MC, 11%; time, 25%; and fq, 17%. Comparing this to the texture C thick puree study [25], with an MC range of 17–20, time 17–22 s, and fq 0.99–1.44, the foods in our study needed less biomechanical work, which further shows the adaptation of oral processing to the textural characteristics of the bolus in older patients, as we previously demonstrated in healthy subjects [19].

The BRS that results from oral processing is critical for the process of safe swallowing. The poor preparation of the bolus could increase the risk of aspiration [16]. The BRS in this study showed a variation of 74% for maximum force, 87% for adhesiveness, and 30% for cohesiveness. The differences in the parameters could be due to bolus factors, as the biomechanical process was quite homogeneous among patients (see Figure 4), as well as the effect of dilution and saliva (in particular the effect of salivary amylase), and also the ingredients and formulations for each dish. Regarding moisture levels and force strength, they were also varied among the participants, though the average moisture data were within the normal range compared to the moisture in the study carried out in Japan in healthy adults in the lingual mucosa. Regarding hand strength force, the averaged force values of this study showed poor mastication capabilities based on the study carried out by Laguna et al., 2016, which correlated a weak hand force with poor mastication capabilities [23].

The safety of swallowing the fork-Mashable dishes varied between them and was less safe compared to the thick purees in our previous paper [25]. This might be due to the complexity of their textural properties. Both the French omelet and the pollock fish, followed by the zucchini omelet, showed the lowest safety of swallowing, possibly due to their high cohesiveness, which was maintained after oral processing and led to the bolus becoming more compacted and difficult to swallow. On the other hand, the red lentils and the potatoes showed a higher safety of swallowing, and this might be due to the increase in adhesiveness after oral processing, along with the cohesiveness. Finally, the stewed turkey showed the highest safety of swallowing, and this is due to this dish having small particles, which lead to them being mixed well and reaching optimum values for both adhesiveness and cohesiveness.

Regarding oral residues, the French omelet and the pollock fish caused the largest residues, probably due to small particles spreading out in the oral cavity and between the teeth during oral preparation. The same applies to the zucchini omelet, as it has the same texture as the French omelet. The rest of the dishes all had high adhesiveness, which let particles stick together in the mouth. Likewise, the pharyngeal residues for both the French omelet and the pollock fish were higher as the dishes with higher cohesiveness were difficult to pass in the pharyngeal phase. The stewed turkey showed the least residue, which might be due to its cohesiveness and adhesiveness coming together, which made it the safest to swallow. Future formulations should focus on achieving textural balance, particularly optimizing the adhesiveness, cohesiveness, and maximum force to improve both the safety and efficacy of swallowing. The palatability analysis showed that both the stewed turkey and the red lentils were the most liked, and both the French omelet and the pollock fish were the least liked, linking this to the safety of swallowing. The PCA shows a different pattern of the dishes and summarizes their results based on their textural properties, biomechanical properties, and V-VST outcomes, as shown. It identified the French omelet and the pollock fish to be the most challenging dishes regarding the safety and efficacy of swallowing due to their compact cohesive bolus. On the other hand, the stewed turkey, red lentils, and potato showed balanced textural properties and, according to this study, the optimal textural values range, which could provide the safest swallowing with a maximum force of 1.0–1.5 N, cohesiveness of 0.4–0.7 N, and adhesiveness of 0.2–0.56 N·s. In addition, the negative values observed before the second compression in these food samples might be due to the probe’s withdrawal phase after the first compression, during which the sample—particularly in its soft, post-oral form—may adhere to the probe. Finally, despite all the dishes in this study meeting the BDA texture E and IDDSI level 6 descriptors, clear variation was observed in their swallowing safety and efficacy. This variation highlights a critical point: compliance with qualitative standards does not guarantee that a food is safe or effective for swallowing. For example, while the stewed turkey demonstrated high safety and efficacy, the French omelet performed poorly in both, despite both dishes falling under the same qualitative classification. These findings reinforce that relying solely on qualitative frameworks like IDDSI and BDA is insufficient to describe the therapeutic properties of texture-modified foods. Therefore, this study advocates for a shift toward quantitative texture characterization—including parameters such as maximum force, cohesiveness, and adhesiveness—which provide more precise, objective, and predictive insights into bolus behavior and its interaction with the patient’s swallowing function.

This study presents some limitations, and in the future, a larger number of dishes and participants should be included. In addition, although it was all soft food, each dish presented different shapes, but the sample size for the texture analyzer considered only the sample weight (30 g). Future work must include a larger variety of dishes and more patient profiles.

## 5. Conclusions

This study demonstrates that the therapeutic effectiveness of texture-modified fork-Mashable diets in patients with mastication and swallowing disorders cannot be determined solely by compliance with BDA texture E or IDDSI level 6 descriptors. Despite meeting these qualitative standards, the dishes showed substantial variation in swallowing safety and efficacy, indicating that such frameworks alone are insufficient for predicting the therapeutic effect of these diets. Key differences in maximum force, cohesiveness, and adhesiveness—especially their changes during oral processing—played a decisive role in bolus formation, clearance, and overall safety. The principal component analysis further confirmed that these quantitative parameters strongly correlate with both therapeutic effectiveness and patient acceptability. Therefore, this study emphasizes moving from qualitative measurements into quantitative textural analyses when making texture-modified foods. This will lead to more accurate results, ensuring both the safety and efficacy of swallowing.

## Figures and Tables

**Table 1:** Gives percentages of the weight in g of the ingredients and the number of kilocalories per recipe portion.

Fork-Mashable	Ingredients	Weight/Recipe (g)	Proteins (g)	Kcal
French omelet	Eggs (70%), potato (25%), onion (2.5%), virgin olive oil (2.5%).	100	11.5	156
Zucchini omelet	Zucchini (25.7%), eggs (30.8%), diced potatoes (41.1%), virgin olive oil (1%), salt (0.9%), and paper (0.5%).	100	2.90	103.62
Stewed turkey	Sliced turkey breast (23.1%), ratatouille (19.4%), water (18.41%), diced potato (13.3%), diced carrot (9.1%), concentrated chicken broth without salt (8.8%), virgin olive oil (5.3%), salt (1.1%), thickener (0.99%), and black pepper (0.5%).	100	13.41	165
Red lentils	Lentils (12.0%), ratatouille (7.2%), concentrated vegetable broth (4.0%), salt (0.7%), extra virgin oil (not provided), diced potato (16.0%), diced carrot (20.0%), water (40.0%), provided), thickener (1.2%).	100	14.5	283
Potato	Potato flakes (91.1%), virgin oil (4.6%), thickener (0.3%), and salt (0.8%).	100	1.06	67.06
Pollock fish	Pollock fish (62.1%), salt (0.6%), olive oil (2.6%), black pepper (0.2%), thickener (0.4%), lemon sauce (33.9%).	100	2.06	146

**Table 2:** Moisture levels, hand grip strength, and age characteristics of participants by sex.

Category	Details	
Patients (n)	20	
Age range	77–94	
Gender	10 women, 10 men	
**Mucus data**	Women (n = 7)	Men (n = 8)
Mucus mean	29.88 ± 6.24	30.91 ± 2.12
Mucus range	15–33	27–33
**Hand grip strength**	Women (n = 7)	Men (n = 8)
Mean (kg)	18.07 ± 3.76	21.75 ± 4.26
Range (kg)	15–21	18–31
**Age**	Women (n = 7)	Men (n = 8)
Mean (years)	85 ± 2.91	86 ± 5.15
Range (years)	82–89	77–94

**Table 3:** Texture-modified diets: pre-oral and post-oral maximum force and percentage of oral processing for selected fork-Mashable dishes.

Fork-Mashable	Pre-Oral Processing		Post-Oral Processing		% Change from Oral Processing
	Mean ± SD		Mean ± SD		
**Maximum force N**					
French omelet	1.1 ± 0.9		0.5 ± 0.1		−51.7
Zucchini omelet	1.7 ± 0.9		1.4 ± 1.0		−16.2
Stewed turkey	1.4 ± 0.9		2.1 ± 0.8		+51.0
Red lentils	1.1 ± 0.5		0.8 ± 0.1		−28.0
Potato	1.0 ± 0.4		0.9 ± 0.2		−7.6
Pollock fish	1.0 ± 0.5		0.6 ± 0.1		−38.0
**Adhesiveness**	N·s	N·m	N·s	N·m	
French omelet	0.25 ± 0.51	(2.5 ± 5.1) × 10^−4^	0.03 ± 0001	(3.0 × 10^−4^) ± (1.0 × 10^−7^)	−88.0
Zucchini omelet	0.02 ± 0.01	(2.0 ± 1.0) × 10^−^⁵	0.27 ± 0.25	(2.7 × 10^−3^) ± (2.5 × 10^−4^)	+92.0
Stewed turkey	0.56 ± 0.53	(5.6 ± 5.3) × 10^−4^	0.98 ± 0.67	(9.8 ± 6.7) × 10^−4^	+75.0
Red lentils	0.99 ± 0.51	(9.9 ± 5.1) × 10^−4^	1.31 ± 0.24	(1.3 × 10^−3^) ± (2.4 × 10^−4^)	+32.0
Potato	0.20 ± 0.42	(2.0 ± 4.2) × 10^−4^	1.53 ± 0.45	(1.5 × 10^−3^) ± (4.5 × 10^−4^)	+86.0
Pollock fish	0.06 ± 0.16	(6.0 ± 1.6) × 10^−^⁵	0.01 ± 0.005	(1.00 × 10^−5^) ± (5.0 × 10^−6^)	−83.0
**Cohesiveness**					
French omelet	0.86 ± 0.05		0.69 ± 0.04		−19.7
Zucchini omelet	0.68 ± 0.10		0.52 ± 0.11		−23.5
Stewed turkey	0.44 ± 0.60		0.53 ± 0.08		+20.4
Red lentils	0.54 ± 0.14		0.75 ± 0.04		+38.8
Potato	0.35 ± 0.17		0.65 ± 0.12		+85.7
Pollock fish	0.66 ± 0.07		0.61 ± 0.05		−7.5

**Table 4:** Mean values of the biomechanical mastication work.

Fork-Mashable	MC	Time (s)	Frequency (Mc/time)
French omelet	31.7 ± 9.68	28.4 ± 5.94	1.12 ± 0.32
Zucchini omelet	29.95 ± 9.4	21.6 ± 8.2	1.39 ± 0.28
Stewed turkey	33.0 ± 13.60	24.57 ± 8.9	1.33 ± 0.29
Red lentils	29.37 ± 12.82	22.87 ± 6.79	1.25 ± 0.31
Potato	29.5 ± 14.29	21.6 ± 9.53	1.36 ± 0.26
Pollock fish	31.0 ± 19.64	24.33 ± 13.00	1.27 ± 0.26

**Table 5:** Mean values as percentages of the acceptability test for all the texture-modified diets regarding their general appearance, texture, smell, and aftertaste.

Attribute	Fork-Mashable	Dislike a Lot % 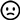	Dislike % 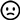	Neither Like nor Dislike 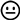	Like % 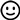	Like a Lot % 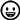
Liking overall	French omelet	0	10	40	40	10
	Zucchini omelet	0	11	44	44	0
	Stewed turkey	0	33	0	50	17
	Red lentils	0	13	38	50	0
	Potato	11	11	44	11	22
	Pollock fish	0	44	22	22	11
Appearance	French omelet	0	10	40	50	0
	Zucchini omelet	11	11	44	33	0
	Stewed turkey	33	17	17	17	17
	Red lentils	0	13	63	25	0
	Potato	0	11	33	56	0
	Pollock fish	0	22	22	56	0
Texture	French omelet	20	0	20	60	0
	Zucchini omelet	0	11	33	56	0
	Stewed turkey	0	50	0	33	17
	Red lentils	0	0	50	38	13
	Potato	0	0	11	78	11
	Pollock fish	0	22	0	78	0
Smell	French omelet	0	10	20	70	0
	Zucchini omelet	0	22	22	56	0
	Stewed turkey	0	17	17	33	33
	Red lentils	0	63	13	25	0
	Potato	11	22	11	56	0
	Pollock fish	0	33	22	33	11
Aftertaste	French omelet	0	30	30	40	0
	Zucchini omelet	0	22	44	33	0
	Stewed turkey	0	17	17	50	17
	Red lentils	0	25	75	0	0
	Potato	0	22	22	56	0
	Pollock fish	0	33	33	33	0

## Data Availability

The data presented in this study are available on request from the corresponding author. ( available due to privacy or ethical restrictions.)

## Supplementary Materials

The following supporting information can be downloaded at: https://www.mdpi.com/article/10.3390/foods14101723/s1, Figure S1: Analysis of the safety of swallowing and efficacy of fork-mashable dishes at different bolus volumes; Figure S2: Different dishes used in this study.
