# Supplementary material for: The Effectiveness of Fork-Mashable Dishes in Managing Older Patients with Mastication and Swallowing Impairments
Source: Foods. 2025 May 13;14(10):1723. doi: 10.3390/foods14101723 (PMC12111229; doi:10.3390/foods14101723)
Supplement: Supplementary file 1 [file foods-14-01723-s001.zip › foods-3621618-supplementary.pdf]

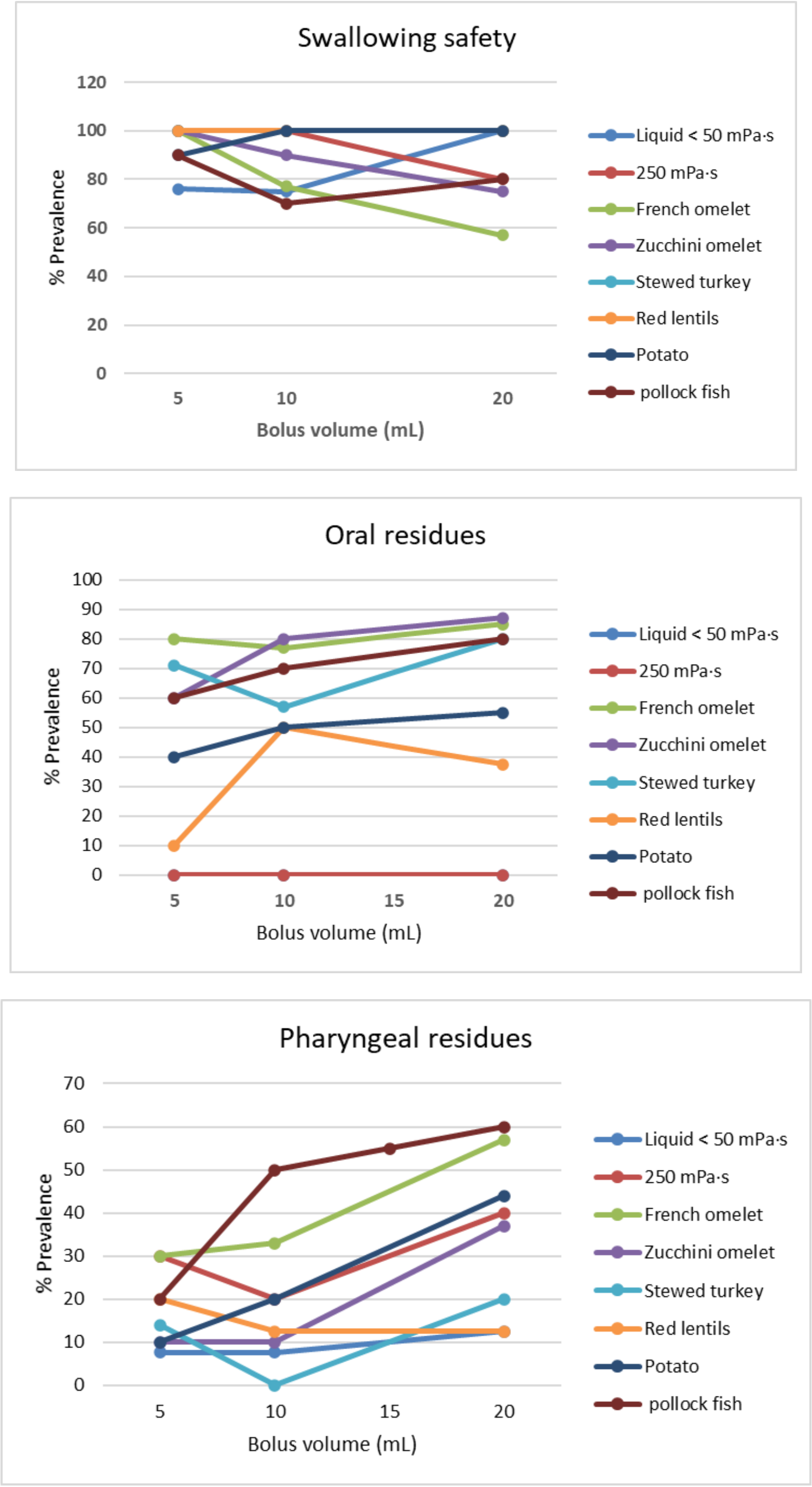

Supplementary Figure S1. Analysis of the safety of swallowing and efficacy of fork-mashable dishes at different bolus volumes.

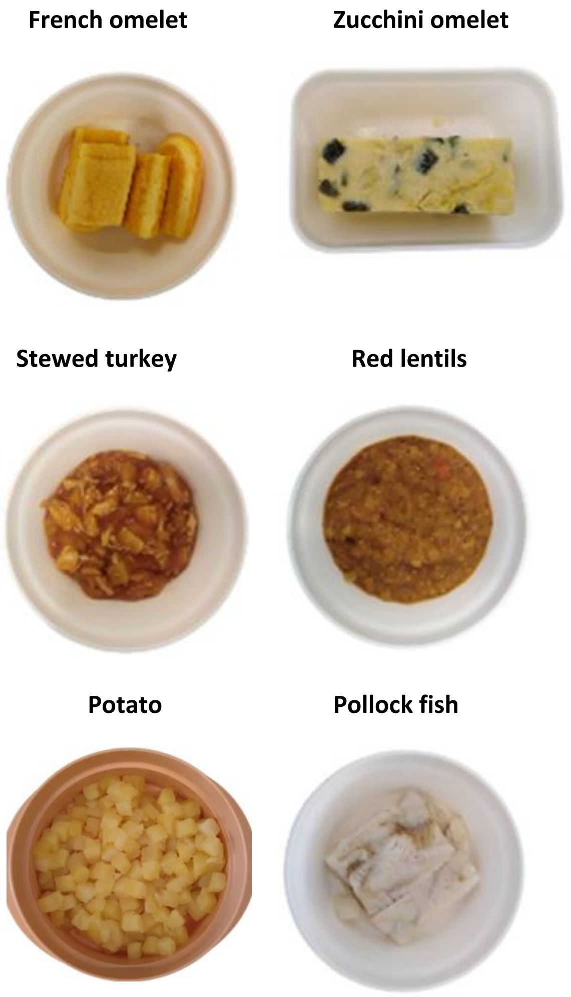

Supplementary Figure S2. Different dishes used in this study
